# Integrative analysis of public ChIP-seq experiments reveals a complex multi-cell regulatory landscape

**DOI:** 10.1093/nar/gku1280

**Published:** 2014-12-03

**Authors:** Aurélien Griffon, Quentin Barbier, Jordi Dalino, Jacques van Helden, Salvatore Spicuglia, Benoit Ballester

**Affiliations:** 1 INSERM, UMR1090 TAGC, Marseille, F-13288, France; 2 Aix-Marseille Université, UMR1090 TAGC, Marseille, F-13288, France

## Abstract

The large collections of ChIP-seq data rapidly accumulating in public data warehouses provide genome-wide binding site maps for hundreds of transcription factors (TFs). However, the extent of the regulatory occupancy space in the human genome has not yet been fully apprehended by integrating public ChIP-seq data sets and combining it with ENCODE TFs map. To enable genome-wide identification of regulatory elements we have collected, analysed and retained 395 available ChIP-seq data sets merged with ENCODE peaks covering a total of 237 TFs. This enhanced repertoire complements and refines current genome-wide occupancy maps by increasing the human genome regulatory search space by 14% compared to ENCODE alone, and also increases the complexity of the regulatory dictionary. As a direct application we used this unified binding repertoire to annotate variant enhancer loci (VELs) from H3K4me1 mark in two cancer cell lines (MCF-7, CRC) and observed enrichments of specific TFs involved in biological key functions to cancer development and proliferation. Those enrichments of TFs within VELs provide a direct annotation of non-coding regions detected in cancer genomes. Finally, full access to this catalogue is available online together with the TFs enrichment analysis tool (http://tagc.univ-mrs.fr/remap/).

## INTRODUCTION

Differences in gene expression programs are believed to play a major role in cell identity and phenotypic diversity in the human body. With the advances of next generation sequencing techniques it became possible to study the genome-wide occupancy maps of transcription factors (TFs) by chromatin immunoprecipitation followed by sequencing (ChIP-seq). The rapid accumulation of ChIP-seq results in data warehouses provides a unique resource of hundreds of occupancy maps.

With the success of the Encyclopedia of DNA elements (ENCODE) project to identify all functional elements in the human genome, the description and annotation of TF binding sites (TFBS) entered a genome-wide era by integrating a hundred TFs. The extent to which the regulatory space is organized along the genome is only starting to unfold with large consortia studies (1–5) but remains largely matter of discoveries (e.g. super enhancers) (6,7). Indeed, recent studies have uncovered hundreds of genomic loci that are co-occupied by multiple TFs in various cell types suggesting the importance and abundance of combinatorial regulation in cells (2,7,8). So far, those diverse regulatory features generated from various studies have not been yet integrated to form a global map of regulatory elements.

Here, we report the complex landscape of TFBS in the human genome. We have constructed a global map of regulatory elements by compiling the genomic localization of 132 different TFs across 83 different cell lines and tissue types based on 395 selected human public (non-ENCODE) data sets. The integration of the genome-wide TFBS allows for the construction of a catalogue of *cis*-regulatory modules (CRMs) of variable complexity.

Specifically, we report a complex map of TFBS increasing by 14% (+439 Mb, +993 421 regulatory features) the human genome regulatory search space compared to the ENCODE catalogue alone. Different studies have proposed to integrate various NGS ChIP-seq data sets but either from a workflow/platform approach (9) or from a quality assessment perspective (10). We performed a detailed comparison of the TFs occupancy map generated from public data against the ENCODE TF catalogue. Both maps are examined at the levels of TFs, TFBS and CRMs scales; finally, both maps are merged allowing the creation of a large catalogue of complex organization of bound regions. In total, after including ENCODE TF data to complement public data, we examined 237 TFs across multiple cell types. This map has been compiled into public tracks in genome browsers allowing users to assess regulatory elements combined with genome annotations in their regions of interests. In addition, the catalogue has been compiled into flat files allowing further computational analyses and is available at http://tagc.univ-mrs.fr/remap/.

Finally, to demonstrate the usefulness of our approach we used this unified catalogue to annotate variant enhancer loci (VELs; H3K4me1 mark) from two cancer cell lines. Our TFs enrichment analyses within those variable regions reveal enrichments of specific TFs those functions are involved in cancer development and proliferation. The work presented here constitutes a solid unification of regulatory regions in the human genome using a systematic integration of public non-ENCODE and ENCODE data. Taken together with our TF enrichment tool it allows for a better annotation of enhancers.

## MATERIALS AND METHODS

### Public/non-ENCODE data sets sources

Public ChIP-seq data sets were extracted from Gene Expression Omnibus (GEO) and ArrayExpress (AE) databases. For GEO, the query *‘(‘chip seq’ OR ‘chipseq’ OR ‘chip sequencing’) AND ‘Genome binding/occupancy profiling by high throughput sequencing’ AND ‘homo sapiens’[organism] AND NOT ‘ENCODE’[project]’* was used to return a list of all potential data sets to analyse, which were then manually assessed for further analyses. Data sets involving polymerases (i.e. Pol2 and Pol3), mutated or fused TFs (e.g. KAP1 N/C terminal mutation, GSE27929) and p300/CBP TFs were excluded.

A *data set* was defined as a ChIP-seq experiment in a given GEO series (e.g. GSE41561), for a given TF (e.g.: ESR1), in a particular biological condition (e.g. MCF-7). Data sets were labelled with the concatenation of these three pieces of information (e.g. GSE41561.ESR1.MCF-7).

We analysed 668 data sets present in the GEO repository starting from July 2008. Those data sets were complemented by 28 data sets present in AE. (Full list of data sets in Supplementary File S1).

### Public ChIP-seq processing

Bowtie 2 (11) with options *–end-to-end –sensitive* was used to align all reads on the human genome (GRCh37/hg19 assembly). Biological and technical replicates for each unique combination of GSE/TF/Cell type or Biological condition were combined after mapping. TFBS were identified using MACS peak-calling tool (12) (version 1.4.1) in order to follow ENCODE ChIP-seq guidelines, with stringent thresholds (*P*-value: 1e-5; enrichment: 10; False discovery rate, FDR: 0.01). An input data set was used when available. Because of the large size of peaks sometimes identified by MACS (up to 62 kb; mean size: 505.2 bp, median size: 377 bp), we used the tool PeakSplitter (13) (version 0.1) to retrieve shorter peaks (up to 4.7 kb; mean size: 350.9 bp, median size: 304) containing summits.

### ENCODE data sets

We used the ENCODE release V3 (August 2013) representing all ENCODE TF ChIP-seq experiments passing quality assessments. This data provides TFBS clustered by factor, based on 690 data sets and 161 TFs (http://hgdownload.cse.ucsc.edu/goldenPath/hg19/encodeDCC/wgEncodeRegTfbsClustered/wgEncodeRegTfbsClusteredV3.bed.gz). We removed 3 TFs (POLR2A, POLR3G, EP300), and renamed 3 TF aliases into official HGNC identifiers (GRp20 into NR3C1, KAP1 into TRIM28, SIN3AK20 into SIN3A) leading to a final list of 155 TFs from ENCODE.

### Quality assessment of public data sets

To assess the quality of public data sets, we computed a score based on the cross-correlation and the FRiP (fraction of reads in peaks) metrics developed by the ENCODE Consortium (14) (Supplementary Figure S2). Two thresholds were defined for each of the two cross-correlation ratios (NSC, normalized strand coefficient: 1.05 and 1.10; RSC, relative strand coefficient: 0.8 and 1.0). Detailed descriptions of the ENCODE quality coefficients can be found at http://genome.ucsc.edu/ENCODE/qualityMetrics.html. We used the phantompeak tools suite (15) (https://code.google.com/p/phantompeakqualtools/) to compute RSC and NSC. The selected cut-off minimum and optimum values for the two ratios are defined in phantompeak tools. NSC values range from a minimum of 1 to larger positive numbers. Note that 1.10 is the critical threshold. Data sets with NSC values much less than 1.10 (<1.05) tend to have low signal-to-noise ratio or few peaks. RSC values range from 0 to larger positive values. Note that 1 is the critical threshold. RSC values much less than 1 (<0.8) tend to have low signal-to-noise ratio, the low scores can be due to failed and poor quality ChIP and/or low read sequence quality. A basal score ranging from 0 to 4 was assigned to each data set corresponding to the number of thresholds it exceeds for NSC and RSC (two thresholds for each score). This basal score was incremented by one if the FRiP is equal or higher than 1%. We observed that data sets having a minimum score of 2 exceeded at least one threshold of RSC or NSC, which are both scores independent of peak calling procedures. Thus, data sets with a final score less than or equal to 1, as well as data sets with fewer than 100 identified peaks were discarded for further downstream analyses. Those data sets were not included in the final catalogue of public peaks available on our resource page (ReMap: http://tagc.univ-mrs.fr/remap/).

### Non-redundant sets of peaks and CRMs definition

To produce a catalogue of discrete, non-redundant binding regions in the genome for each TF, we used BedTools (16) (version 2.17.0) to merge overlapping peaks (with at least 1 bp overlap) identified in different data sets for similar TFs. Public and ENCODE binding sites were combined before the overlap. The summit of resulting peaks was defined as the average position of the summits of merged peaks. Similarly, to obtain the CRMs in the genome, overlapping peaks of all TFs in the catalogue were merged using BedTools. Regions bound by several TFs are called CRMs, whereas regions bound by only one TF are labelled as singletons.

### Genomic localization

To localize regulatory regions (Peaks and CRMs) in the genome, we used CEAS (17) (version 0.9.9.7). Each region was assigned to a genomic localization such as intergenic, intronic, exonic, 5′- or 3′-UTR or promoter (−3 kb upstream to TSS) based on human RefSeq annotation (GRCh37/hg19). Repartition of CRMs around TSSs was analysed after extraction of the TSSs from Ensembl genes (Ensembl release v67).

### Comparison with genomic resources

Public sets of genes, protein coding genes and regulatory elements (Vista enhancers and ORegAnno) were downloaded from the Ensembl genome portal (Ensembl release v67) using BioMart and from the UCSC table browser tool (hg19 assembly). Promoters were defined as −2 kb/+1 kb from transcription start sites (TSS). ENCODE DNaseI clusters (V2, January 2013) were downloaded from UCSC (http://hgdownload.cse.ucsc.edu/goldenPath/hg19/encodeDCC/wgEncodeRegDnaseClustered/wgEncodeRegDnaseClusteredV2.bed.gz). We employed BedTools for overlap analyses allowing 1 bp overlap.

### Motif discovery

The RSAT (Regulatory Sequence Analysis Tools) suite was used for *de novo* motifs analyses in non-redundant binding sites of each TF. The RSAT program *peak-motifs* (18) with the options -*-markov auto –minol 6 –maxol 8 –merge_lengths –2str –scan_markov 3* was run to detect overrepresented words and dyads, which were then compared against known motifs from JASPAR core vertebrates database (version November 2013). ENCODE motifs were extracted from FactorBook (19), and JASPAR motifs from the JASPAR core vertebrates database (20) (version 5.0_alpha).

### Conservation scores

For each of the 237 TFs present in our catalogue we assessed the DNA constraint for each base pairs 1 kb around the summit of each peak. Conservation scores were obtained from the Ensembl Compara database (21) release v67. Genomic Evolutionary Rate Profiling (GERP; (22)) score was used to calculate the conservation of each nucleotide in multi-species alignment. The multiple whole genome alignment used to derive GERP score is the 20-way amniota vertebrates Enredo-Pecan-Ortheus (EPO) alignment.

### Network analyses

The overlap of non-redundant binding sites for each couple of TFs was computed using IntervalStats tool (version 1.01) (23). For each peak in the query set of binding sites, IntervalStats computes a *P*-value of the overlap of this peak with the reference set of binding sites. A *P*-value threshold was defined as 0.05 to identify significant overlapping peaks with the reference. Each TF was used both as query and as reference in each couple of TFs, forming an asymmetric matrix of the percentages of significant overlapping peaks between two TFs (Supplementary Figure S7). For each TF, a list of strongly and moderately specific TFs was determined by identifying outliers based on the percentages of significant overlapping peaks. Outliers were defined as TFs that have a percentage exceeding 1.5 and 3.0, respectively, for moderately and strongly specific TFs, the interquartile range above the 75th percentile of percentages (Supplementary Figure S8). A network was generated using Gephi tool (24) (version 0.8.2) with all TFs that co-localized with at least one other TF. The size of nodes is correlated with the number of interactors (i.e. co-localized TFs) for each TF. The weight of edges corresponds to strong (thick line) or moderate (thin line) specificity of the co-localization of TFs, and colour represents the percentages of significant overlapping peaks between the two TFs. The graph was partitioned into subnetworks using an algorithm developed by Blondel *et al*. (25) and implemented in Gephi, with options *randomize, use edge weights* and a resolution of 0.51.

### VELs

Two sets of VELs were analysed in this study, from CRC (colorectal cancer) and MCF-7 (breast cancer) cell lines. First, the list of CRC gained and lost VELs described in Akhtar-Zaidi *et al*. (26) were downloaded from GEO (GSE36204). For downstream analyses hg18 VELs were converted into hg19 coordinates using the *liftover* tool of UCSC (http://genome.ucsc.edu/cgi-bin/hgLiftOver) with default parameters returning 2604 gained and 3448 lost CRC VELs.

On the other hand, to create the MCF-7 VELs we applied the same procedures as described in the materials and methods of Akhtar-Zaidi *et al*. on H3K4me1 of MCF-7/MCF-10A data sets produced by Choe *et al*. (27). Sequencing data were downloaded from the Sequence Read Archive (SRA045635) of the National Center for Biotechnology Information. Read alignments, peaks calls and VEL detection were all performed using the exact same procedure as described in Akhtar-Zaidi *et al*. We obtained a set of 3163 gained and 2791 lost MCF-7 VELs using a normal distribution with a standard deviation of 1 to model the uniformly distribution of reads. To assess the MCF-7 VELs detection procedure we also generated VELs with relaxed thresholds (SD = 0.75, 5295 for Gained, 5087 for Lost) and report those result in Supplementary Figure S9. IntervalStats tool was used to compute and identify significant overlapping TFBS with VELs. TF enrichments in VELs were calculated via a hypergeometric test.

We have extracted RNA-seq data for MCF-7 and MCF-10A cell lines, respectively, from GEO (GSE48213). We compared gene expression (FPKM, fragments per kilobase per million mapped reads) of genes associated with gained, lost and control VEL. Genes were associated with VEL regions using the GREAT tool (great.stanford.edu). Control loci are H3K4me1 loci identified in common between the MCF-7 and MCF-10A cell lines.

### Public access and annotation tool

Our catalogues of public peaks and public + ENCODE peaks are publically available either as a track in the UCSC genome browser and also with extra analyses at http://tagc.univ-mrs.fr/remap/. An annotation tool has been deployed, available on the website, allowing user to query their regions of interests against our catalogue of TFBS to identify enrichments of TFs. The entire catalogues are also available to download.

## RESULTS

Our overall approach is to analyse ChIP-seq data sets for TFs from public repositories in order to provide a comprehensive map of regulatory elements. To do this we first extracted and processed all available public data sets, which were merged with the ENCODE TF catalogue to produce an extensive repertoire of regulatory regions in the human genome (Flowchart Supplementary Figure S1). We then used this regulatory catalogue to identify genome-wide co-localized TFs and TFs enriched in VEL present in colon cancer and breast cancer cell lines. We further present an online tool providing a full web access to this catalogue and allow users to annotate their genomic regions with regulatory elements.

### Integration of various ChIP-seq data sources

We initially selected 696 data sets from public repositories, such as the GEO (28) and AE (29). To ensure consistency and comparability, each of these ChIP-seq data sets was re-analysed from the raw reads, through our ChIP-seq analysis pipeline which included read mapping, peak calling and quality assessment based on ENCODE quality criterions. Those quality criterions determined whether a data set would be included or not for further downstream analyses or excluded. Indeed, ChIP-seq data sets are not all equal in terms of quality (10), these differences come from the rapid development of next-generation sequencers and sequencing techniques, but also from experimental improvements in library preparations and quality.

To address this variability of ChIP-seq quality we used two metrics independent of peak calling based on ENCODE ChIP-seq guidelines and practices. First, we used the normalized strand cross-correlation coefficient (NSC) which is a ratio between the maximal fragment-length cross-correlation value and the background cross-correlation value, and the relative strand cross-correlation coefficient (RSC), a ratio between the fragment-length cross-correlation and the read-length cross-correlation (14). Both values are stringent metrics for assessing signal-to-noise ratios in a ChIP-seq experiment and have been correlated with high quality data sets (Encode guidelines (14)). Those ChIP-seq quality analyses are in accordance with current assessments of disparate published ChIP-seq data sets (10). However, on top of those scores based on alignments quality we added two metrics based on peak properties: the FRiP and a useful but simple first-cut metric, the number of peaks in the data set. By using these four scores we were able to evaluate ChIP-seq data and filter out low quality data sets (Supplementary Figure S2).

We initially collected, analysed and assessed 696 raw TF ChIP-seq data files in various conditions from Illumina sequencers and available in public data warehouses (GEO, AE). After applying our quality filters based on these four ChIP-seq metrics we retained 395 data sets (54.3%) from 135 different GEO series (GSEs) involving 132 TFs (Supplementary Table S1). More precisely, we define here a ‘*data set*’ as a ChIP-seq experiment in a given GEO series (e.g. GSE41561), for a given TF (e.g. ESR1), in a particular biological condition (i.e. cell line, tissue type, disease state or experimental conditions; e.g. MCF-7). Data sets were labelled with the concatenation of these three pieces of information (e.g. GSE41561.ESR1.MCF-7). A data set may correspond to several replicates when multiple samples were done in the same GEO series, for the same TF under identical biological conditions. TFBS were computed following our peak-calling pipeline using MACS tool as described in detail in the method section. We identified 8.9 million ChIP-seq peaks bound by TFs in the human genome across all data sets. The complete data sets (mapped reads and called peaks) are available from our online companion (http://tagc.univ-mrs.fr/remap/).

**Figure 1.**
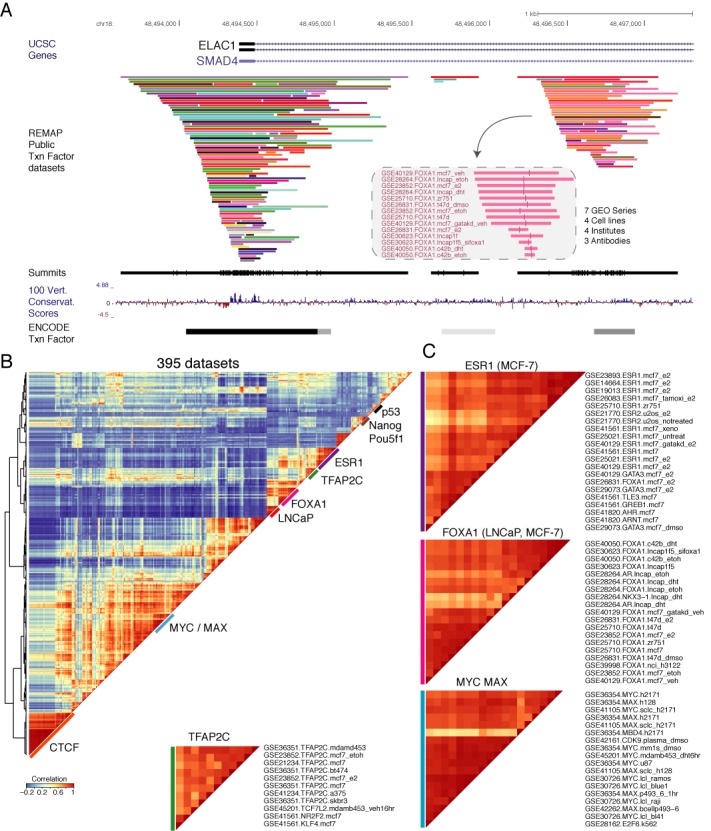
ChIP-seq binding pattern of 395 data sets. (**A**) A genome browser example of complex ChIP-seq binding patterns of the 395 data sets at the SMAD4/ELAC1 promoters, and a detailed view of the redundant peaks for a FOXA1 site. The following genome tracks correspond to the ChIP-seq peak summits (black vertical lines), the 100 vertebrates conservation track from UCSC and the condensed ENCODE TF bindings. (**B**) Co-binding correlation patterns of the 395 data sets are clustered and shown as a heatmap with blue to red indicating low to high correlations for each co-localized data sets. Co-binding relationships between TFs and cell types across all data sets are observable. Co-localization clusters are highlighted with coloured bars and (**C**) some clustered data sets are shown in details (e.g. ESR1 in MCF-7 cells).

Binding sites for similar TFs but produced from different data sets mapped onto the genome revealing tight and dense co-localization of sites in many regions of the genome (Figure 1A). This clustering of binding sites from different data sets is illustrated as an example within the first intron of SMAD4 and ELAC1 genes by a FOXA1 site recapitulated by 14 data sets. Those data sets correspond to 7 different GEO series (GSE), 4 different cell lines (MCF-7, ZR-75-1, LNCaP, C4-2B), 4 different institutes (the Cancer Research UK Cambridge Institute, the Genomic Institute of Singapore, the University of Helsinki, the Washington University School of Medicine) and finally 3 different antibodies (Abcam ab5089, ab23738, ab4124). Interestingly, it can be noted that the summits (vertical bars) of the peaks aggregate closely from each other. Those aggregations of FOXA1 summits are an illustration of what is globally observed across millions of peaks on the genome. This FOXA1 example is a simple demonstration that global integrative analyses of ChIP-seq data sets can improve the detection of TFBS. Indeed, the presence of a binding site in different data sets at this position for FOXA1 reinforces the possibility that this specific TF is binding the DNA *in vivo* at this genomic location.

To further investigate whether our re-analysed public data sets correlated with each other we generated a correlation matrix based on the genomic localization of peaks (Figure 1B). The 395 data sets were clustered on the basis of co-binding correlations. Heatmap colours correspond to Pearson's correlation coefficients of low (blue) to high (red) range of co-binding affinity. Consistently, we observe that data sets are generally clustered by similar TFs, or similar cell lines. Several dense clusters with high binding co-occurrences are detected within this correlation matrix. The most striking clusters of TFs with highly correlated binding profiles are clusters regrouping most of p53, NANOG/POU5F1, ESR1, TFAP2C, MYC/MAX and CTCF. Some of those clusters tend to be also enriched in specific cell lines LNCaP and MCF-7 (Figure 1C). The ESR1 cluster regroups 8 out 10 GEO series and consists mainly of the MCF-7 cell type with different experimental conditions. It can be noted that the CTCF insulator forms a tight cluster containing all the CTCF data sets in this analysis (Figure 1B, orange). This indicates that most CTCF bindings are shared across all different ChIP-seq experiments regardless of the cell types. This result complements current findings about CTCF (30,31) where there is little difference of CTCF binding between cell types (3). Taken together these clusters indicate that a consistent analytic approach of multiple data sets can recapitulate binding sites and reveal that some TFs bind similar locations in the genome. Although the integration of independent ChIP-seq studies is challenging, the overlap observed between peaks for similar TF validate this approach in order to improve the annotation of regulatory regions in the human genome.

### Identifying and dissecting CRMs

**Figure 2.**
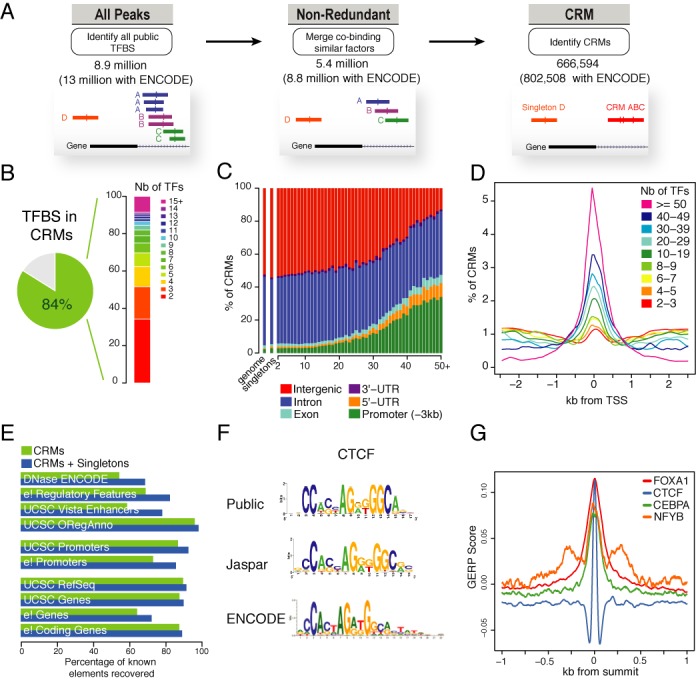
ChIP-seq peaks and CRMs. (**A**) A schematic diagram of the three types of regulatory regions: all peaks, non-redundant peaks and CRMs. Peaks for similar TFs overlapping the same regions were merged into single peaks defined as non-redundant. For each genomic region bound by at least two different TFs, those bindings were regrouped into CRMs. (**B**) Proportion of single and combined binding sites observed after identification of CRMs. The vertical barplot correspond to proportion of CRMs found in combinatorial binding categories across all identified CRMs. (**C**) Genomic distribution of single or combined binding sites in six different genomic regions. The percentage of binding sites in each category is shown on the vertical axis, for the overall genome, singletons and each combinatorial binding complexity from 2 to 50+ TFs. (**D**) Distribution of CRMs at TSS (±2.5 kb) for increasing levels of combinatorial binding complexity from 2 to 50+ TFs. (**E**) Proportion of our regulatory catalogue covering different types of genomic features. Percentages of elements recovered are shown for CRMs only (green) and both CRMs and singletons (blue). (**F**) The WebLogo position weight matrix diagrams for CTCF identified across the diverse databases, showing subtle position-specific differences. (**G**) DNA sequence constraint around the peak summits of FOXA1, CTCF, CEBPA, NFYB were plotted by observed-expected GERP scores (22).

**Figure 3.**
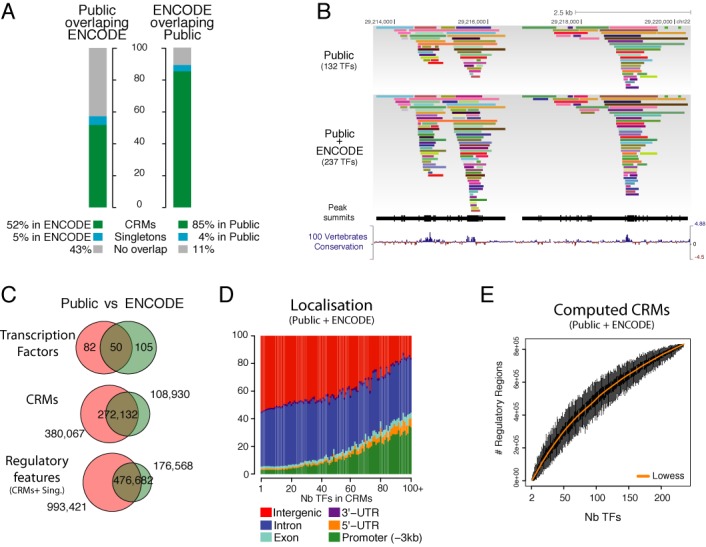
Comparison with ENCODE and integration with public data. (**A**) Comparisons of public regulatory regions versus ENCODE regions. The vertical barplots correspond to the proportion of TFBS from the integrative analysis of public data that can be recapitulated in the ENCODE CRMs and singletons. ‘No overlap’ corresponds to potential novel regulatory regions. Overlap analyses are performed both ways. (**B**) A genome browser example of binding patterns from public data only, and complemented patterns with the public and ENCODE merge. The following genome tracks correspond to the ChIP-seq peak summits (black vertical lines) and the 100 vertebrates conservation track from UCSC. (**C**) Venn diagrams of TFs, CRMs and regulatory features (CRMs and singletons) between the public set and ENCODE. (**D**) Genomic distribution of single or combined binding sites in six different genomic regions. The percentage of binding sites in each category is shown on the vertical axis for singletons and each combinatorial binding complexity from 2 to 100+ TFs. (**E**) Saturation analysis of the ReMap data with increasing numbers of TFs. The plot is generated from the merge of both public and ENCODE TFBS catalogues. This plot illustrates the saturation of CRMs identified by TF ChIP-seq as additional factors are analysed across the multi-cell integrative analysis. We calculate CRMs counts across the genome from an increasing number of TFs randomly selected. The distribution of CRMs counts for 100 TFs selection is plotted as a boxplot on the *x*-axis. We continue to do this for all incremental steps up to and including all TFs. A lowess line smoothing the medians of the CRMs count is highlighted in orange.

Our initial catalogue of 8.9 million binding sites identified across all data sets includes overlapping sites bound by similar TFs immunoprecipitated in various conditions and therefore do not reflect the total number of discrete binding regions across the genome. To address this redundancy between data sets we merged binding sites of similar TFs, resulting in a catalogue of 5.4 million sites which we define as non-redundant peaks (Figure 2A). As non-redundant peaks are made of at least two or more peaks for a given factor, we addressed whether our method would modify the summit location for those non-redundant peaks. We observe a mean variation of 37 bp between the summits of the non-redundant peaks set and the individual summits of peaks they are made of (Supplementary Figure S3). These narrow summit variations across peaks of similar TF justify the use of a method to merge them into non-redundant peaks. The genomic organization of our occupancy map reveals dense co-localizations of sites forming tight clusters of heterogeneous binding sites with variable TFs complexity (Figure 1A). Those clustering patterns of TFBS along the genome were defined as CRMs by merging overlapping peaks into 666 594 CRMs. Among our catalogue of 5.4 million non-redundant TFBS, 84% can be incorporated into CRMs. About half of CRMs contains 2 or 3 TFs and span a few hundred base pairs, whereas the other half corresponds to regions binding multiple TFs creating complex regulatory elements spanning few kilobases (Figure 2B). For example, out of our CRMs set 8.4% is composed of complex combination of TFs containing 15 or more TFs (Figures 1A, 2B and 3B). These highly complex CRMs are mainly found in promoters and around TSSs reminding previously defined highly occupancy targets regions frequently associated with promoters (32,33). Our large TFBS catalogue leads to 666 594 regulatory sequence elements where about 50% form complex CRMs.

Since single TFBS have been shown to positively correlate with gene density across the genome (34), we examined the relationship between CRM complexity and their genomic localization (Figure 2C and D). We observe that half of singletons and small CRMs fall within gene bodies and in close gene proximity (Promoters, UTRs). However, as CRMs complexity increases, their localization shows a clear preference for promoters. The enrichment of CRMs at proximal promoters is consistent with previous findings (35). However, as CRMs complexity increases the proximal promoters preferential occupancy is done in detriment to intergenic/distal regions. Interestingly, we observe that on average 2% of Singletons and CRMs are localized within exons which support the possible role of DNA binding protein on restricting transcripts diversity (5). Here, we show that proximal promoters can potentially act as platforms of extremely complex and dense TF bindings. To further address this we also investigated the TF bindings around the TSSs (±2.5 kb) (Figure 2D). We observe a positive relationship between dense binding and TSS with complex CRMs preferentially positioned at TSS. Taken together those results indicate that core promoters can potentially attract a large set of transcriptions factors and co-factors. Interestingly, it also highlights the presence of intergenic CRMs attracting 10–30 TFs possibly acting as distal or remote enhancers. These results agree with findings where distal enhancers are associated with active chromatin marks in a cell-type-specific way (31), whereas promoters tend to be constitutively occupied in multiple cell lines.

To determine the accuracy of our enhancer catalogue we next identified the fraction of major genomic resources of known elements that are covered by our data. We first analysed genome-wide set of regions that are likely to be involved in gene regulation, such as accessible chromatin and regulatory resources. We recovered 70% of ENCODE DNaseI hypersensitive sites (50% CRMs only), 80% of Ensembl regulatory build based, 78% of Vista Enhancers and 95% of ORegAnno annotations. In addition to regulatory regions, we also compared our catalogue with various sources of annotated promoters. We recovered 90% of UCSC promoters and 85% of Ensembl promoters. Finally, we compared our catalogue with human gene build from three major gene annotation resources: UCSC, Ensembl and RefSeq. We recovered 90% of UCSC genes, 90% of Ensembl coding genes and 70% of all Ensembl genes. The 10–30% of genes not recovered by our catalogue are in majority non-coding genes and pseudo-genes, either not bound by TFs or bound by TFs not yet present in our catalogue (Supplementary Table S2). As previously shown a large fraction of annotated genes are recovered as 40% of our catalogue fall within gene bodies mainly introns, but also exons and UTRs (Figure 2C). The above evidences indicate that our catalogue correctly identifies most known annotated genomic elements and annotated enhancers from major public resources.

To test whether this catalogue of TFBS could consolidate DNA motifs found in current databases or provide alternative/variable motifs we performed *de novo* motif discovery (Figure 2F). When available we examined Jaspar (36) and FactorBook/ENCODE (19) DNA motifs for a given TF and compared those with our top motifs discovered by RSAT *peak-motifs* tool (18). An example for the CTCF DNA logo is shown in Figure 2F where subtle bases variations can be observed as well as consolidation of unchanged bases in the motif. It is well established that ChIP-seq provide an excellent way to perform *de novo* motif analyses as DNA motifs are enriched around peaks summits helping the detection of the DNA motifs for those TFs (14,37). Our catalogue of TFBS can contribute to refine or consolidate TF DNA matrices currently present in motif databases.

It has been shown that DNA motifs are highly conserved across species (37) thus the DNA sequence are under a selective pressure. We thus examined for each TF the sequence conservation around all non-redundant peaks. This analysis used the GERP differential scores where expected and observed scores refer to the number of expected and observed substitutions in the sequences (weighted by the branch lengths of the tree). For four selected TFs (FOXA1, CTCF, CEBPA, NFYB) we observe an increase of DNA constraints under the summit positions of the ChIP-seq peaks among the vertebrate species (Figure 2G). Interestingly, NFYB DNA constraints describe a trimodal distribution possibly as a result of the nature of the nuclear TF Y forming a trimeric complex (with NFYA and NFYC) binding to the DNA with high specificity and affinity. Those DNA constraints observations can be observed globally on the genome browser with 100 vertebrate phastcons conservation track (Figures 1A and 3B). Taken together those analyses show that we have correctly identified most binding sites coming from public sources, that those sites regroup into 666 594 CRMs of various complexities and are possibly identifying new regulatory regions in the human genome.

### Complementing ENCODE TFBS data set

To address whether this catalogue would identify new regulatory regions within the genome, we analysed our data by comparing our results with the reference annotation of regulatory regions in the human genome, the latest ENCODE catalogue of DNA elements (4). The ENCODE catalogue consists of 155 TFs that allowed the community to gain a better understanding in the annotation of regulatory regions in the human genome. We first assessed whether both catalogues would overlap or complement each other. We observe that ENCODE regulatory elements overlap by 89% the public catalogue, but only 57% of the public regulatory elements overlap with ENCODE (Figure 3A). This suggests that our catalogue based on public ChIP-seq data is complementary to ENCODE regions, and that the number of regulatory regions in the human genome may be greater than anticipated. This overlap of ENCODE versus public regulatory elements is illustrated as a genome browser track on Figure 3B, where we observe ENCODE TFBS complementing public CRMs. When comparing those two catalogues, only 50 TFs are found in common between the two studies, 105 TFs are specific to ENCODE and 82 TFs are specific to the public catalogue (Figure 3C). We also found that 272 132 CRMs are in common in both catalogues.

To improve the accuracy of our catalogue and to complement the annotation of TF bound elements in the human genome we merged both ENCODE TFBS set and the public catalogue leading to a large repertoire of 8.8 million TFBS generated from 237 TFs (Figure 2A). This allowed the generation of 802 508 CRMs with complex modules binding 50 or more TFs. The number of CRMs containing 15 TFs or more increases from 8.4% using public data only to 13.9% when merging both catalogues. Looking at their genomic localization we observe that complex CRMs concentrate at promoters in detriment to intergenic regions, agreeing previous findings where enhancers tend to be cell-type specific, and promoters constitutively bound (Figure 3D). Also by merging both catalogues we observe that the human genome regulatory search space is increased by 14% (+439 Mb) when comparing with the ENCODE genomic coverage only.

Finally, to address whether the regulatory regions detected in the human genome would reach a plateau we computed CRMs based on the random selection of increasing number of TFs (100× ranging from 2 to 237 TFs) (Figure 3E). We observe that the number of CRMs is not finite in the genome, as the computation of CRMs from random permutations of TFs does not reach a plateau. Each additional TF continued to show additional CRMs rather reaching saturation of regulatory regions in the genome. As our catalogue contains diverse cell types, our result suggests that many cell-specific binding sites potentially exist in diverse cell types. Those conclusions are consistent with results obtained by ENCODE for TFs across different cell lines (38), with open chromatin elements defined by FAIRE and DNaseI (4), and with the 8 millions of TF sites as described by DNAseI footprinting (5).

### Cooperating TFs network

**Figure 4.**
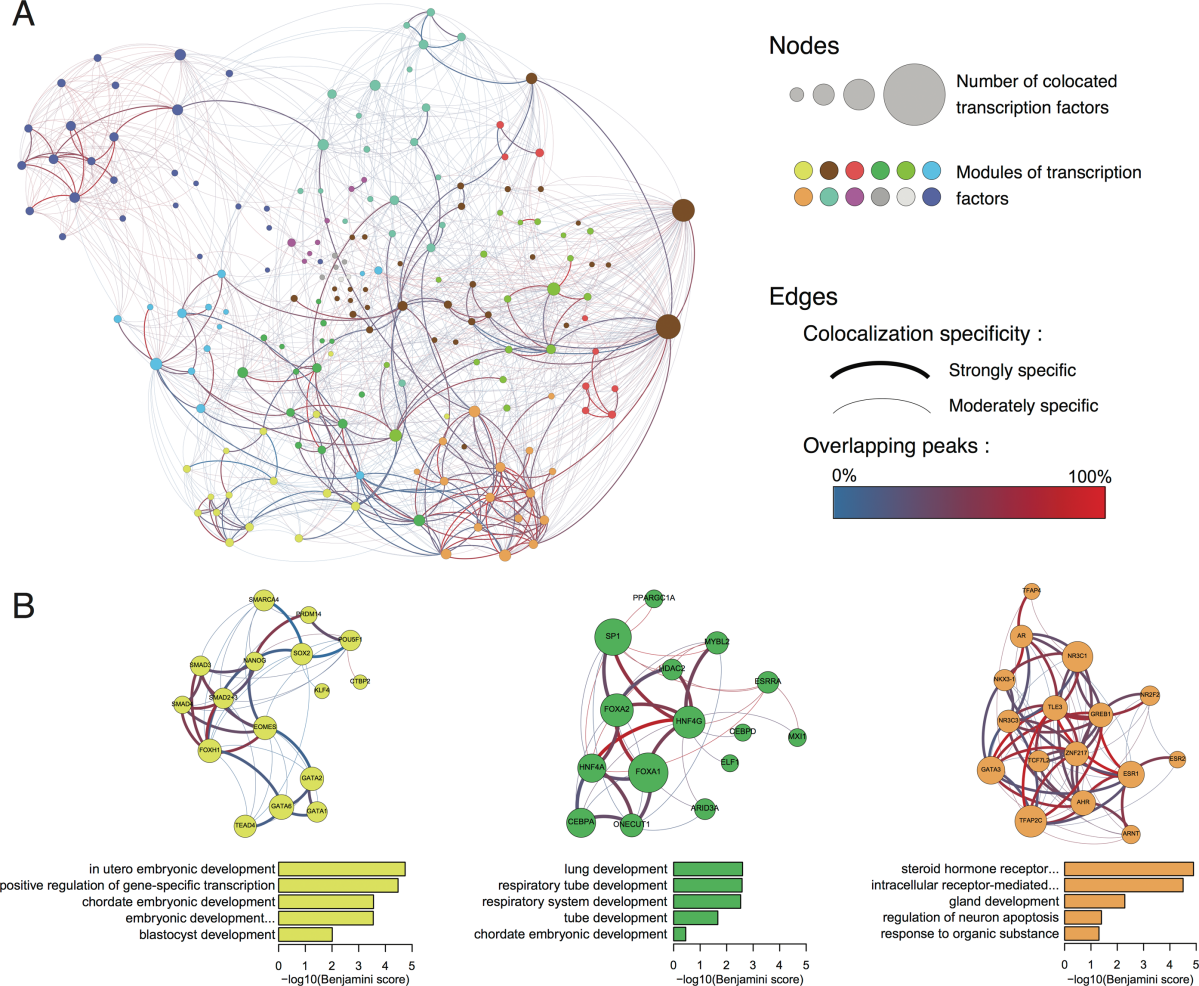
Network representations of TFs co-localization across the genome. (**A**) In this filtered TF co-localization network, nodes indicate individual TFs and colours indicate subnetworks identified by applying a partitioning algorithm; edge colours depict the percentages of overlap between TFBS and weights the co-localization specificity between two TFs. Overlapping binding sites were computed using IntervalStats tool and co-localization specificity was determined by identifying outliers based on the percentages of significant overlapping sites. (**B**) Highlighted subnetworks of highly connected and strongly specific TFs with functional annotations. Barplots represent Gene Ontology *Biological Process* enrichments calculated by DAVID (*x*-axis = −log10 Benjamini score).

In a specific cellular context, genes are regulated by several TFs that cooperate to increase or decrease the gene expression as demonstrated in recent work (4,39–42). This is especially the case for tissue-specific genes involving regulatory elements that bind cocktails of specific TFs. To identify these specific combinations of TFs, we generated a co-localization network based on the overlap of binding sites observed in our catalogue. We used IntervalStats tool to compute significant overlaps between binding sites of each pair of TFs and to calculate the percentage of overlapping sites between factors. Based on this percentage we identified strong and moderate co-localization specificity of TFs (see Materials and Methods section). Gephi tool was used to create and visualize the network containing 181 co-localized TFs (Figure 4A). In this network, the weight of the edges represents how specifically two TFs are associated together, whereas the colour indicates their percentage of overlapping binding sites. Although the network reveals that all TFs are globally highly interconnected, we can distinguish clusters of strongly specific TFs. To highlight these clusters of TFs, we partitioned the network into 12 subnetworks in different colours (Figure 4B and Supplementary Figure S4) using an algorithm developed by Blondel *et al*. and implemented in Gephi. To further understand the biological role of these groups of TFs, we annotated them using the DAVID online tool (Figure 4B). The first subnetwork (Figure 4B, yellow subnetwork) is annotated with embryonic development terms as it contains the POU5F1 (Oct4), SOX2 and NANOG TFs. The second TFs subnetwork (green) reveals affinities with lung development functions. It contains FOXA1 and FOXA2 both being co-expressed in respiratory epithelial cells throughout lung morphogenesis (27), as well as SP1 those expression regulates lung tumour progression (28). Finally, a TFs cooperation subnetwork (orange) is functionally enriched in hormone receptor annotations as it contains factors, such as AR, ESR1, NR3C1 and NR3C3. Here, we show that using our global occupancy map of 237 TFs we can highlight groups of cooperating TFs having specific functional enrichments. Finding functional annotations for groups of TFs is a challenging task as all proteins are associated with Gene Ontology terms, such as *TF* or *DNA binding domain*. However, the TFs subnetworks identified based on co-localization data only reveal relevant biological signatures. It can be noted that our co-localization network show a global interplay of TFs across the genome, however, the extent to which some TFs act as master regulators is yet to be discovered. Some studies started to describe how TFs could be ubiquitous or tissue/function specific (43) but the complexity of TF interplay is yet to be unfolded.

### Application to VEL in cancer

**Figure 5.**
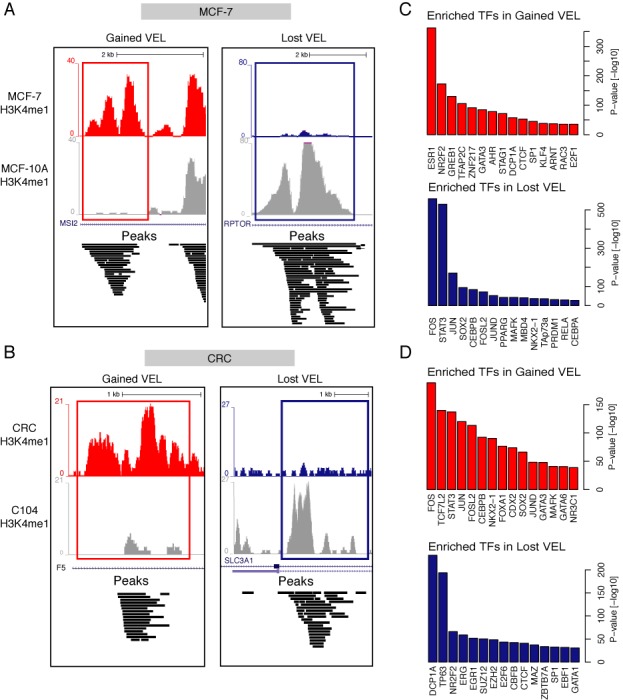
Specific TFs signature in VELs. (**A**) UCSC browser views of H3K4me1 profile of a normal mammary epithelial cell line (MCF-10A) and a breast cancer cell line (MCF-7), illustrating an example of a gained (left in red) and a lost (right in blue) VELs. (**B**) Similar view of H3K4me1 profile for a primary CRC (CRC V400) and a normal colon epithelium crypt (C104). (**C**) TFs specifically enriched within regions defined as gained or lost VELs in MCF-7 and CRC (**D**) cell lines.

As a direct application of our catalogue we examined the binding of TFs within gained and lost enhancer loci in primary CRC cell lines relative to normal colon epithelium crypts as published by Akhtar-Zaidi (26). We applied the same strategy to identify VELs from H3K4me1 mark in human breast cancer cell line (MCF-7) relative to normal mammary epithelial cell line (MCF-10A). Using sets of gained and lost enhancers in both cancers we asked whether those enhancers would enrich for specific TFBS biologically related to these cancers. First, we observed that 95–98% of VELs contained one or more TFBS (Supplementary Table S3) with a clear positioning of binding sites at the center of those loci (Figure 5A and B and Supplementary Figure S5). When crossing our TF occupancy catalogue against those loci, we identified enrichments of very specific TFs present in gained or lost VELs, respectively.

For breast cancer, we report enrichment for ESR1 DNA bindings in gained enhancer loci in MCF-7 cell line relative to MCF-10A cell line (Figure 5C), as well as NR2F2 which encodes a member of the steroid thyroid hormone receptor, involved in apoptosis and increased proliferation (44). Both of those TFs have been actively studied for their role in breast cancers development. Our analysis allows for the selection of potential enhancers/target-genes couples potentially involved in pathological processes leading to breast cancer. We observe an enrichment of FOS and JUN present in lost VELs, meaning that the binding of those two TFs can lead to an AP-1 complex loss in MCF-7 enhancers. The AP-1 complex controls a number of cellular processes including differentiation, proliferation and apoptosis. This loss of DNA binding in MCF-7 enhancers could indeed deregulate normal cellular processes.

Regarding colorectal VELs, we find TP63 to be highly enriched within lost VELs (Figure 5D), it is a member of p53 family of TFs and has been shown to suppress tumorigenesis and metastasis (45). Interestingly, SUZ12 and EZH2 are enriched in lost VELs. SUZ12 is part of the polycomb group (PcG) family of proteins forming a complex with EZH2. The PcG is thought to play a role in the epigenetic repression systems. Those proteins are involved in maintaining the transcriptional repressive state of genes, leading to transcriptional repression of the affected target gene. The loss of DNA bindings for TP63, SUZ12 and EZH2 in those enhancers could activate genes involved in tumorigenesis and metastasis. Interestingly, DCP1A also know as TF SMIF is the most enriched TF in this group, its protein is expressed the most in colon RKO cancer cells according to the MaxQuant database (46). For gained VELs in colon cancer we observe an enrichment of TCF7L2, also known as TCF4, involved in many cancer types (47). A frameshift mutation of TCF7L2 has been shown to be implicated in CRC (48). Interestingly, FOS and JUN are present in gained enhancers in colon cancer, leading to implication in the differentiation, proliferation and apoptosis of colon cancer cells. Furthermore, the enrichment of STAT3 binding provides further evidence of regulation of cell growth and apoptosis. The different TF enrichments found for FOS and JUN within MCF-7 and CRC cell lines can be explained by the different biological context, but also by the fact that different combinations of AP-1 dimers can regulate a breadth of cellular events (49).

Taken together the observed enrichments of TFs within VELs in CRC and MCF-7 are involved in key processes, such as differentiation, proliferation or apoptosis, a clear signature of cancer. Their presence in either gained or lost enhancers provides a clue whether those processes are activated or repressed. Indeed, we support previously published evidence of gene expression changes associated with VELs (26). By analysing RNA-seq gene expression data in MCF-7/MCF-10A we observe up-regulated genes associated with gained VELs and down-regulated genes with lost VELs (Supplementary Figure S6). Our H3K4me1 enhancer analysis in cancers coupled with our regulatory catalogue allow the fine dissection of potential key regulatory players and regions in those cancers.

### A web resource for exploring the TFBS catalogue and annotating regions

The results presented here provide an informative annotation for 13 million TFBS coming from public sources and ENCODE data, which include in total 237 TFs across 83 diverse cell lines. This catalogue is a great source of information for dissecting site-specific TF bindings (e.g. FOXA1 in Figure 1A) or for genome-wide binding analyses. To facilitate the exploration of the regulatory elements by the scientific community, we created an online resource called ReMap (http://tagc.univ-mrs.fr/remap/) to display information about TFs (description, classification, external references), peaks and data sets (quality assessment, read mapping and peak calling statistics, conservation score under the peaks). This web resource enables data (full catalogue of TFBS and binding sites for specific TFs) to be downloaded in BED or FASTA format for input into other analysis pipelines, such as motifs discovery tools (18). Genomic tracks containing all peaks (from public data only or from public and ENCODE merging) are available as tracks in the UCSC Genome Browser for browsing and visual exploration. We also developed a tool to allow the annotation of genomic regions provided by users. Those regions are compared against the ReMap catalogue returning statistical enrichments of TFs present within input regions compared to random expectations. It thus becomes possible to study bindings of specific TFs overrepresented in those regions.

## DISCUSSION

In this study, we present an extensive catalogue of TFBS forming complex architecture in the human genome revealed by the integration of public ChIP-seq data. We observe that the regulatory binding landscape of our genome is only starting to unravel as 8.8 million of non-redundant sites are being detected with public non-ENCODE and ENCODE data sets involving a total of 237 TFs. This high number of bound regions is concordant with the 8.4 million distinct short sequence elements from DNase I footprinting found across 41 cell types (4). Indeed, one striking result is that 794 megabases (Mb, 25.3%) of the genome is occupied by our catalogue of bound DNA-binding proteins. The latest ENCODE ChIP-seq analyses estimate 636 336 binding regions covering 355 Mb (11.3%) of the genome across all cell types (4). We observe a similar number of 666 594 CRMs by integrating public non-ENCODE ChIP-seq data only; however, this number increases up to 802 508 CRMs when merging the ENCODE repertoire.

Our analyses provide two evidences. First, there is more complexity in regulatory regions than initially thought. Indeed, the number of binding sites in the genome does not double, but increases by 63% (from 5.4 to 8.8 M) when adding 105 TFs specific to ENCODE. However, the number of detected TF DNA-bound regions does not seems to be yet a finite number of regions in the human genome as the CRMs number does not reach a plateau (Figure 3E). Interestingly, compared to the amount of TFBS the number of CRMs remains low, giving an opportunity to identify complex occupancy regions defined as CRMs containing multiple TFBS. Indeed, we observed CRMs with highly complex TFs combination. Those large CRMs could be affiliated as super-enhancers recently described to control cell identity as they are associated with key genes controlling cell state (6). Our results lay the groundwork for further global annotation and fine dissection of super-enhancers in the genome.

Our catalogue provides a global view of all detected TF binding in a wide variety of cellular context. In our study we observe a high fraction of TF binding in promoters in a multi-cellular context, as our catalogue aggregates multiple biological sources. At the level of histone modifications, Shen *et al*. (3) have found in mouse that the occupancy of enhancers by H3K4me1 is still the most tissue-specific, whereas they observe that H3K4me3 mark occupies most RefSeq promoters in multiple tissues. Looking at the co-binding patterns of TFs in human, Wang *et al*. (42) have described cell-type-specific binding of sequence-specific TF. They observed that genes specifically expressed in a cell line are often associated with a greater occurrence of nearby TF binding in that cell line. They also show a correlation between the amount of TF binding in a given cell line and their genes expression. Thus, high concentration of observed binding sites potentially increase TF interactions, as cell-type-specific interactions among TFs can play a critical role in different gene expression (50). Finally, variations of the chromatin accessibility between cell types have to be taken into account for downstream analyses (51). However, our catalogue gives a first clue of genomic binding of 237 TFs in specific locations in the genome across multiple biological conditions.

One of the major advantages of this catalogue is the ability to compare occupancy maps across different data sets for a single factor. This comparison of bound regions for the same factor allows for direct evaluation of binding sites (Figure 1A). While quality assessments and metric scores of ChIP-seq data have been applied to select and evaluate ChIP-seq data sets (10,52), the analysis of global integration of public ChIP-seq occupancy maps while including ENCODE has not been studied in depth. Even with an automated pipeline the heterogeneity of those public non-ENCODE data sets necessitates a time-consuming manual curation coupled with an understanding of the analyses description provided. Also, the blind application of quality metrics without biological context should be taken with cautions (10), hence, the benefit of annotating redundant binding sites across multiple cell types and studies. In their work Marinov *et al*. demonstrate that a mediocre ChIP-seq data set can possibly receive a high QC score, or as opposite have a low QC score for a good data set. However, our findings from analysing 395 ChIP-seq data sets together with data from ENCODE suggest that a global integration may provide an overall landmark in the annotation of TF occupancy maps in the human genome.

A logical application to this catalogue would be to investigate this new regulatory search space like exons-based approaches in order to characterize the effect of SNPs on regulatory regions at a global scale (53,54). For instance, in our study we validated the added genomic value of this catalogue by interrogating our regulatory map to analyse VEL in colorectal and breast cancer cell lines. We dissected those VELs for specific enrichments of TFs giving new insights into the possible implications of key regulators role in those cancers.

How many of these regulatory regions have multiple roles under different cellular environments and how this catalogue can be classified between gene regulatory elements and enhancers is only starting to unfold (6,55,56). However, our catalogue is a multi-cell map currently limited to the cell types and TFs stored in data warehouse. The integration of more ChIP-seq data may reveal other major occupancy maps, complement current CRMs or affine CRMs composition. How many of these detected regulatory elements contribute to gene expression is an open question. Indeed, the next major step in the understanding of gene regulation will reveal physical and direct links between those regulatory regions and their target genes, but more globally between the landscape of bound elements and the rest of our genome. Techniques, such as chromatin conformation capture (3C, 4C, 5C) (57), Hi-C (58) and ChIA-PET (59,60), started to provide the first clues of those interactions. However, how much of those regions are truly functional is still an open question.

In summary, we have shown that integration of public non-ENCODE ChIP-seq data sets provide a large catalogue of complex regulatory elements organized along the genome. In combination with ENCODE, this regulatory map complement and complexify current findings. Although new data sets are constantly added to repositories we believe that this first large regulatory factor occupancy resource may help in better understanding the regulation processes in human.

## SUPPLEMENTARY DATA

Supplementary Data are available at NAR Online.

*Authors Contributions*: Conceived and designed the study: A.G., B.B.; Analysed the data: A.G., B.B.; Data collection: A.G., J.D.; Conceived the website and the annotation tool: Q.B.; Statistical analyses and guidance: A.G., Q.B., J.V.H.; Wrote the paper: A.G., J.V.H., S.S., B.B. All authors read and approved the final manuscript.

SUPPLEMENTARY DATA
