# Supplementary material for: Integrative analysis of public ChIP-seq experiments reveals a complex multi-cell regulatory landscape
Source: Nucleic Acids Res. 2014 Dec 3;43(4):e27. doi: 10.1093/nar/gku1280 (PMC4344487; doi:10.1093/nar/gku1280)
Supplement: SUPPLEMENTARY DATA [file supp_43_4_e27__index.html]

Integrative analysis of public ChIP-seq experiments reveals a complex multi-cell regulatory landscape — Integrative analysis of public ChIP-seq experiments reveals a complex multi-cell regulatory landscape — Integrative analysis of public ChIP-seq experiments reveals a complex multi-cell regulatory landscape — SUPPLEMENTARY DATA 

# Integrative analysis of public ChIP-seq experiments reveals a complex multi-cell regulatory landscape

## SUPPLEMENTARY DATA

**Files in this Data Supplement:**

- SUPPLEMENTARY DATA
