# Supplementary material for: Integrative analysis of public ChIP-seq experiments reveals a complex multi-cell regulatory landscape
Source: Nucleic Acids Res. 2014 Dec 3;43(4):e27. doi: 10.1093/nar/gku1280 (PMC4344487; doi:10.1093/nar/gku1280)
Supplement: SUPPLEMENTARY DATA [file supp_gku1280_nar-02520-met-g-2014-File007.pdf]

SUPPLEMENTAL INFORMATION:

Integrative analysis of public ChIP-seq experiments reveals a complex multi-cell regulatory landscape.

SUPPLEMENTAL TABLES 2

Table S1 List of the 132 transcription factors available in the public catalogue. 2
Table S2 Biotypes of genes not recovered by our catalogue. 5
Table S3 Overlap statistics of VELs from CRC and MCF-7 cell lines with our regulatory catalogue (peaks and CRMs). 5

SUPPLEMENTAL FIGURES 6

Figure S1 Analysis and pipeline flowchart 6
Figure S2 Quality assessment of public datasets 7
Figure S3 Variation of summits of merged peaks relative to the average summit of non-redundant peaks. 8
Figure S4 Clusters of highly interconnected and strongly specific colocalized TFs. 9
Figure S5 Normalized distance from TFBS to the center of Variant Enhancer Loci. 10
Figure S6 Breast cancer VELs correlate with gene expression variations. 10
Figure S7 Matrix of the percentages of significant overlapping peaks for each pair of TFs. 11
Figure S8 Determination of the colocalisation specificity of two TFs (example for SOX2). 12
Figure S9 Transcription factors specifically enriched within lenient sets of gained and lost VELs in MCF-7. 13

SUPPLEMENTAL TABLES

Table S1 List of the 132 transcription factors available in the public catalogue.

	HGNC symbol	HGNC description
**1**	AFF4	AF4/FMR2 family, member 4
**2**	AHR	aryl hydrocarbon receptor
**3**	AR	androgen receptor
**4**	ARNT	aryl hydrocarbon receptor nuclear translocator
**5**	ATRX	alpha thalassemia/mental retardation syndrome X-linked
**6**	BCL6	B-cell CLL/lymphoma 6
**7**	BCOR	BCL6 corepressor
**8**	BRD2	bromodomain containing 2
**9**	BRD3	bromodomain containing 3
**10**	BRD4	bromodomain containing 4
**11**	BRF2	BRF2, RNA polymerase III transcription initiation factor 50 kDa subunit
**12**	CBFB	core-binding factor, beta subunit
**13**	CBX3	chromobox homolog 3
**14**	CDK8	cyclin-dependent kinase 8
**15**	CDK9	cyclin-dependent kinase 9
**16**	CDX2	caudal type homeobox 2
**17**	CEBPA	CCAAT/enhancer binding protein (C/EBP), alpha
**18**	CEBPB	CCAAT/enhancer binding protein (C/EBP), beta
**19**	CTCF	CCCTC-binding factor (zinc finger protein)
**20**	CTNNB1	catenin (cadherin-associated protein), beta 1, 88kDa
**21**	DCP1A	decapping mRNA 1A
**22**	E2F1	E2F transcription factor 1
**23**	E2F4	E2F transcription factor 4, p107/p130-binding
**24**	E2F6	E2F transcription factor 6
**25**	E2F7	E2F transcription factor 7
**26**	ELF5	E74-like factor 5 (ets domain transcription factor)
**27**	ELL2	elongation factor, RNA polymerase II, 2
**28**	EOMES	eomesodermin
**29**	ERG	v-ets avian erythroblastosis virus E26 oncogene homolog
**30**	ESR1	estrogen receptor 1
**31**	ESR2	estrogen receptor 2 (ER beta)
**32**	ETS1	v-ets avian erythroblastosis virus E26 oncogene homolog 1
**33**	ETV1	ets variant 1
**34**	EZH2	enhancer of zeste homolog 2 (Drosophila)
**35**	FLI1	Fli-1 proto-oncogene, ETS transcription factor
**36**	FOXA1	forkhead box A1
**37**	FOXH1	forkhead box H1
**38**	FOXM1	forkhead box M1

**39**	FOXP1	forkhead box P1
**40**	GABPA	GA binding protein transcription factor, alpha subunit 60kDa
**41**	GATA1	GATA binding protein 1 (globin transcription factor 1)
**42**	GATA2	GATA binding protein 2
**43**	GATA3	GATA binding protein 3
**44**	GATA6	GATA binding protein 6
**45**	GATAD1	GATA zinc finger domain containing 1
**46**	GPS2	G protein pathway suppressor 2
**47**	GREB1	growth regulation by estrogen in breast cancer 1
**48**	GTF2B	general transcription factor IIB
**49**	HNF4A	hepatocyte nuclear factor 4, alpha
**50**	HSF1	heat shock transcription factor 1
**51**	IKZF1	IKAROS family zinc finger 1 (Ikaros)
**52**	JUND	jun D proto-oncogene
**53**	KLF4	Kruppel-like factor 4 (gut)
**54**	MAX	MYC associated factor X
**55**	MBD4	methyl-CpG binding domain protein 4
**56**	MED12	mediator complex subunit 12
**57**	MEF2C	myocyte enhancer factor 2C
**58**	MEIS1	Meis homeobox 1
**59**	MYC	v-myc avian myelocytomatosis viral oncogene homolog
**60**	NANOG	Nanog homeobox
**61**	NCOA1	nuclear receptor coactivator 1
**62**	NCOR1	nuclear receptor corepressor 1
**63**	NCOR2	nuclear receptor corepressor 2
**64**	NFKB1	nuclear factor of kappa light polypeptide gene enhancer in B-cells 1
**65**	NFYA	nuclear transcription factor Y, alpha
**66**	NFYB	nuclear transcription factor Y, beta
**67**	NIPBL	Nipped-B homolog (Drosophila)
**68**	NKX2-1	NK2 homeobox 1
**69**	NKX3-1	NK3 homeobox 1
**70**	NOTCH1	notch 1
**71**	NR2F2	nuclear receptor subfamily 2, group F, member 2
**72**	NR3C1	nuclear receptor subfamily 3, group C, member 1 (glucocorticoid receptor)
**73**	NR3C3	progesterone receptor
**74**	ONECUT1	one cut homeobox 1
**75**	ORC1	origin recognition complex, subunit 1
**76**	PHF8	PHD finger protein 8
**77**	POU5F1	POU class 5 homeobox 1
**78**	PPARG	peroxisome proliferator-activated receptor gamma
**79**	PRAME	preferentially expressed antigen in melanoma
**80**	PRDM14	PR domain containing 14
**81**	RAC3	ras-related C3 botulinum toxin substrate 3 (rho family, small GTP

		binding protein Rac3)
**82**	RAD21	RAD21 homolog (S. pombe)
**83**	RB1	retinoblastoma 1
**84**	RBPJ	recombination signal binding protein for immunoglobulin kappa J region
**85**	RELA	v-rel avian reticuloendotheliosis viral oncogene homolog A
**86**	REST	RE1-silencing transcription factor
**87**	RNF2	ring finger protein 2
**88**	RUNX1	runt-related transcription factor 1
**89**	RUNX1+3	runt-related transcription factors 1 and 3
**90**	RUNX1T1	runt-related transcription factor 1; translocated to, 1 (cyclin D-related)
**91**	RUNX2	runt-related transcription factor 2
**92**	RXRA	retinoid X receptor, alpha
**93**	SETDB1	SET domain, bifurcated 1
**94**	SFMBT1	Scm-like with four mbt domains 1
**95**	SMAD1	SMAD family member 1
**96**	SMAD2+3	SMAD family members 2 and 3
**97**	SMAD3	SMAD family member 3
**98**	SMAD4	SMAD family member 4
**99**	SMARCA4	SWI/SNF related, matrix associated, actin dependent regulator of chromatin, subfamily a, member 4
**100**	SMC1A	structural maintenance of chromosomes 1A
**101**	SMC4	structural maintenance of chromosomes 4
**102**	SNAPC1	small nuclear RNA activating complex, polypeptide 1, 43kDa
**103**	SNAPC4	small nuclear RNA activating complex, polypeptide 4, 190kDa
**104**	SNAPC5	small nuclear RNA activating complex, polypeptide 5, 19kDa
**105**	SOX2	SRY (sex determining region Y)-box 2
**106**	SP1	Sp1 transcription factor
**107**	SPI1	spleen focus forming virus (SFFV) proviral integration oncogene
**108**	STAG1	stromal antigen 1
**109**	STAT1	signal transducer and activator of transcription 1, 91kDa
**110**	STAT4	signal transducer and activator of transcription 4
**111**	STAT5A	signal transducer and activator of transcription 5A
**112**	STAT5B	signal transducer and activator of transcription 5B
**113**	SUZ12	SUZ12 polycomb repressive complex 2 subunit
**114**	TAF2	TAF2 RNA polymerase II, TATA box binding protein (TBP)-associated factor, 150kDa
**115**	TAF3	TAF3 RNA polymerase II, TATA box binding protein (TBP)-associated factor, 140kDa
**116**	TAL1	T-cell acute lymphocytic leukemia 1
**117**	TAp73a	tumor protein p73 (isoform a)
**118**	TAp73b	tumor protein p73 (isoform b)
**119**	TBL1	transducin (beta)-like 1X-linked
**120**	TCF12	transcription factor 12
**121**	TCF3	transcription factor 3
**122**	TCF4	transcription factor 4

**123**	TCF7L2	transcription factor 7-like 2 (T-cell specific, HMG-box)
**124**	TFAP2C	transcription factor AP-2 gamma (activating enhancer binding protein 2 gamma)
**125**	TFAP4	transcription factor AP-4 (activating enhancer binding protein 4)
**126**	TLE3	transducin-like enhancer of split 3 (E(sp1) homolog, Drosophila)
**127**	TP53	tumor protein p53
**128**	TP63	tumor protein p63
**129**	VDR	vitamin D (1,25- dihydroxyvitamin D3) receptor
**130**	YY1	YY1 transcription factor
**131**	ZNF143	zinc finger protein 143
**132**	ZNF76	zinc finger protein 76

Table S2 Biotypes of genes not recovered by our catalogue.

UCSC RefSeq genes	%
**protein-coding genes**	26.89
**non-coding genes**	73.11

UCSC genes	%
**protein-coding genes**	23.67
**non-coding genes**	76.33

Ensembl genes	%
**protein-coding genes**	2.95
**non-coding genes**	54.73
**pseudogenes**	39.52
**others**	2.80

Table S3 Overlap statistics of VELs from CRC and MCF-7 cell lines with our regulatory catalogue (peaks and CRMs).

Overlapping data	CRC	MCF-7
***All* VELs with peaks**	98% (5975/6052)	95% (5683/5954)
***Gained* VELs with peaks**	99% (2597/2604)	91% (2894/3163)
***Lost* VELs with peaks**	97% (3378/3448)	99% (2789/2791)
***All* VELs with CRMs**	94% (5723/6052)	92% (5492/5954)
***Gained* VELs with CRMs**	99% (2580/2604)	85% (2710/3163)
***Lost* VELs with CRMs**	91% (3143/3448)	99% (2782/2791)

SUPPLEMENTAL FIGURES

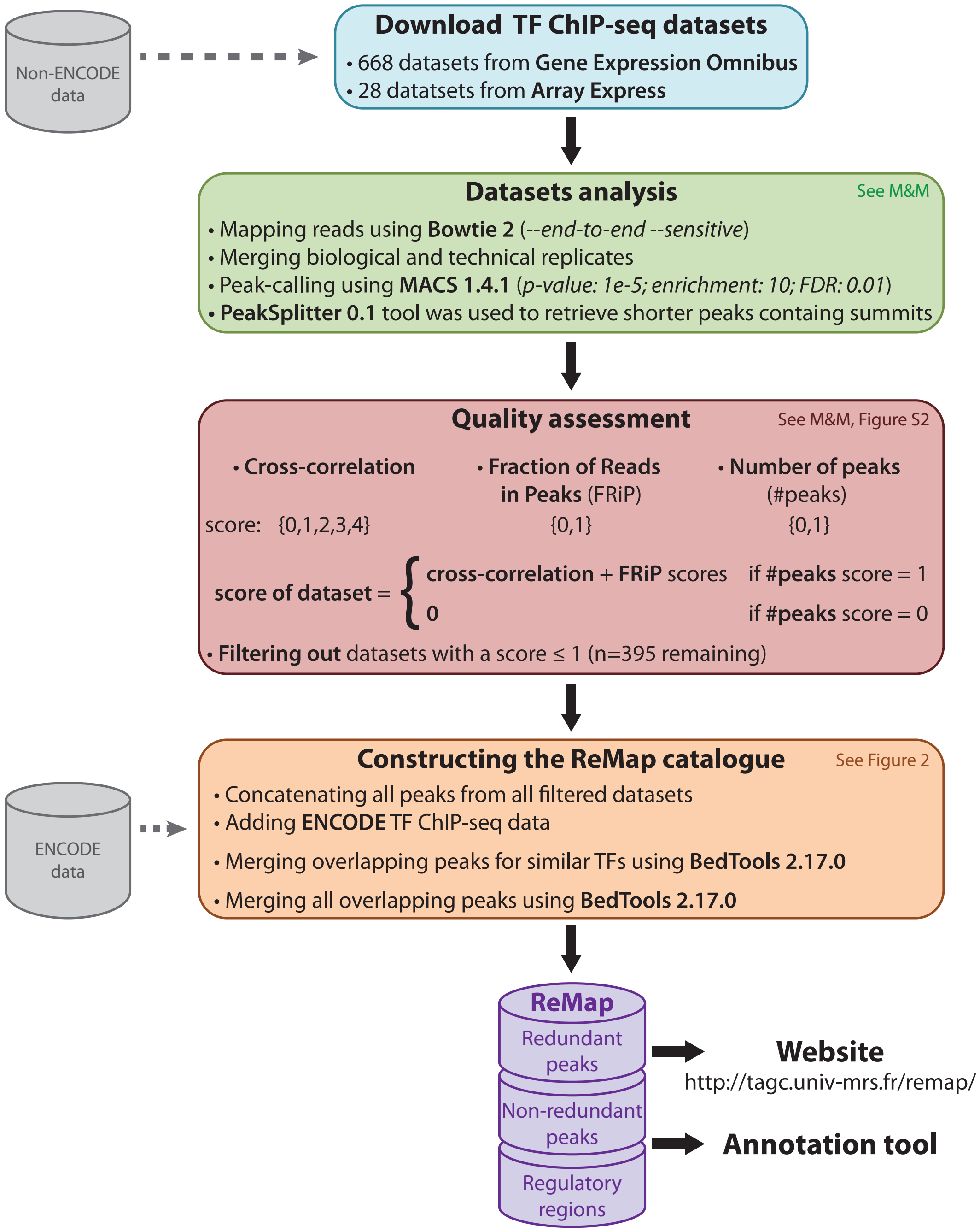

Figure S1 Analysis and pipeline flowchart

Schematic overview of our analysis pipeline used to process public TF ChIP-seq data, assess their quality following approved guidelines, and finally construct the ReMap catalogue while including ENCODE TF ChIP-seq data.

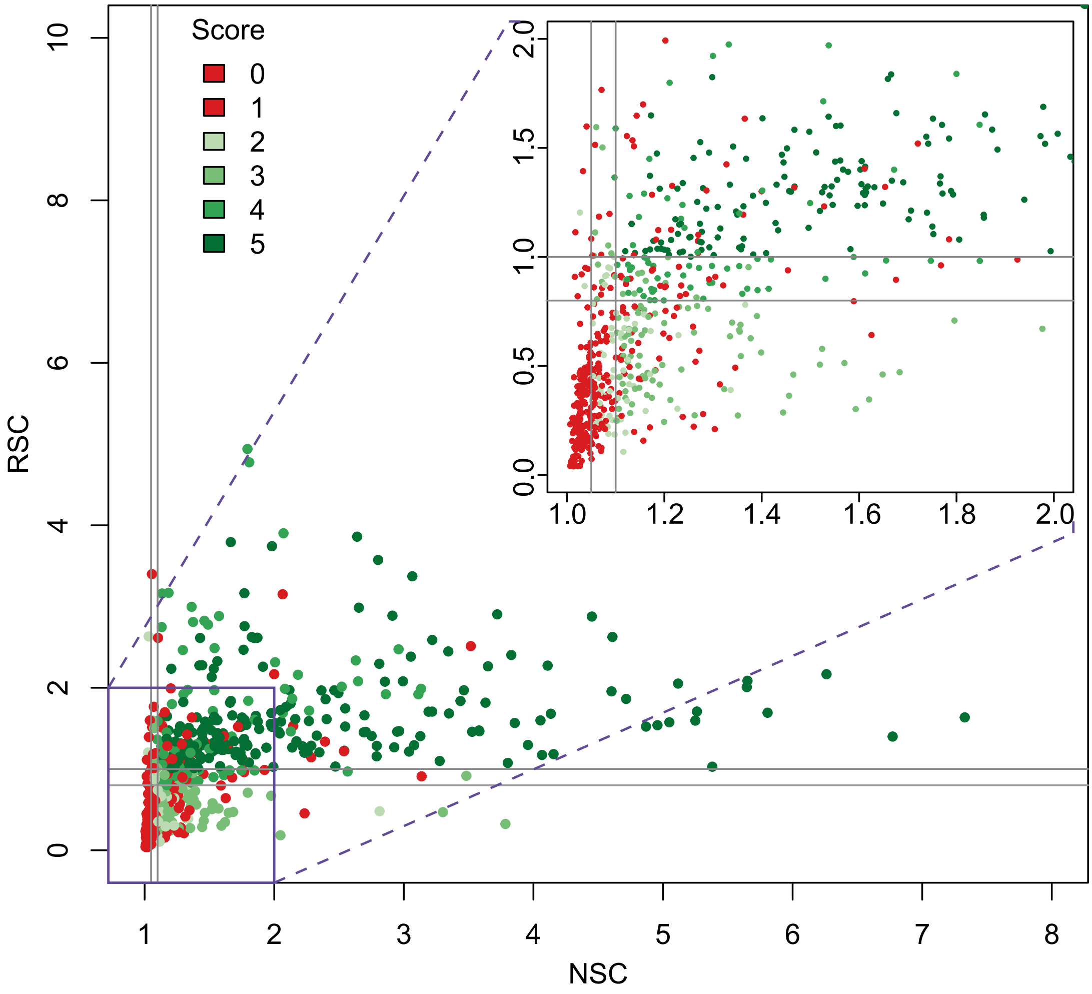

Figure S2 Quality assessment of public datasets

Here each analysed dataset (n=696) is represented by a colored dot according to its assigned score. Indeed, to assess the quality of public datasets, we computed a score based on the cross-correlation and the FRiP (fraction of reads in peaks) metrics developed by the ENCODE Consortium and the phantompeak tools. This score is computed as follow. Two thresholds based on ENCODE studies were defined for each of the two cross-correlation ratios (Grey lines; Normalized Strand Coefficient: 1.05 and 1.10 on the x-axis; Relative Strand Coefficient: 0.8 and 1.0 on the y-axis, see M&M for details). A basal score ranging from 0 to 4 was assigned to each dataset corresponding to the number of thresholds it exceeds for NSC and RSC (2 thresholds for each score). Finally, this basal score was incremented by one if the FRiP is equal or higher than 1%. We observe that datasets having a minimum score of 2 exceed at least one threshold of RSC or NSC, which are both scores independent of peak calling procedures. Thus for our analyses, datasets having a score less than or equal to 1 (red dots), as well as datasets with fewer than 100 identified peaks were discarded for further downstream analyses. Reds dots within accepted thresholds are datasets with less than 100 peaks. Datasets with scores greater to 1 were kept for further analyses (green dots, n=395).

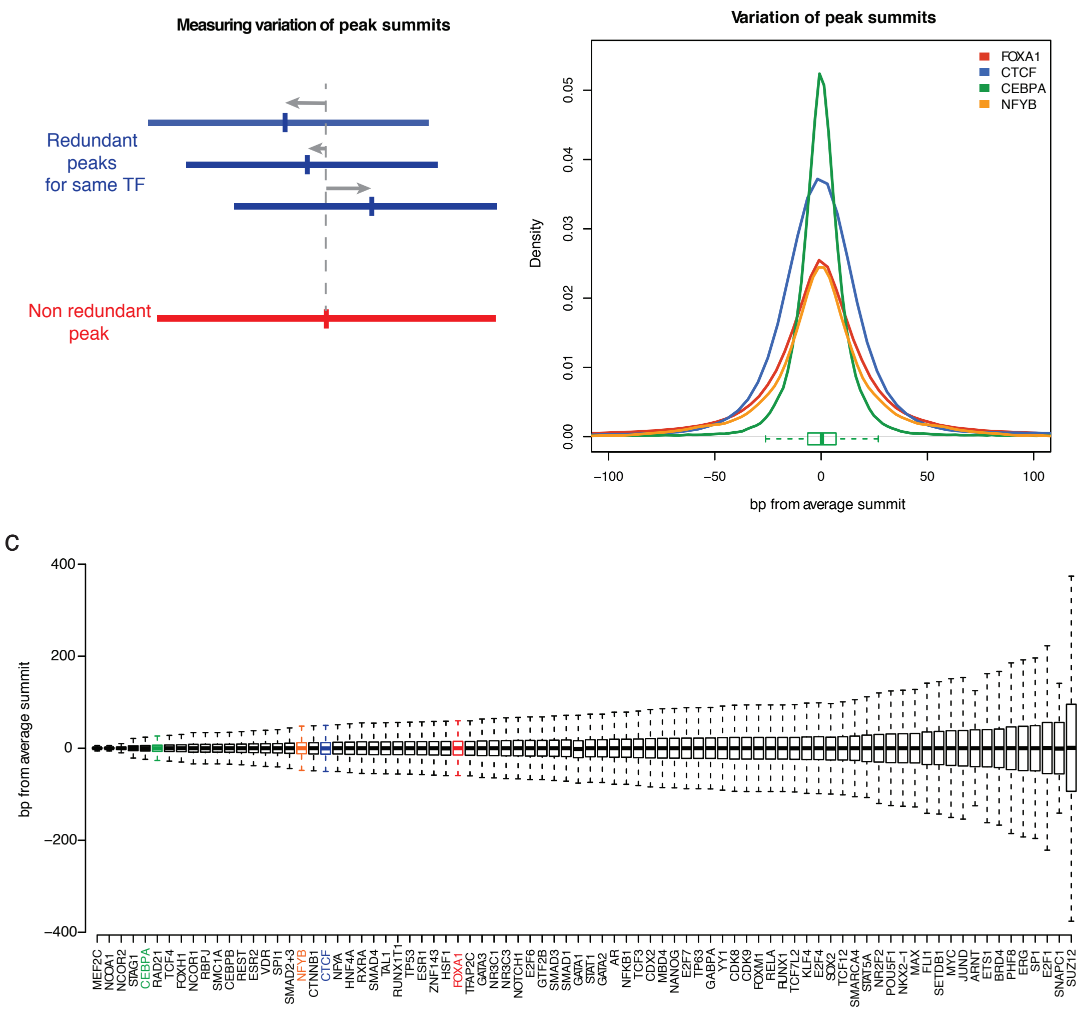

Figure S3 Variation of summits of merged peaks relative to the average summit of non-redundant peaks.

(**A**) Schema explaining how non-redundant peaks are computed, and how summits variation is measured. (**B**) Distribution of distances between non-redundant peaks summit and the summits of all peaks they are made of, for four selected factors (FOXA1, CTCF, CEBPA and NFYB) similar to the main Figure 2g. (**C)** Boxplot showing the summits variation for all factors having more than 100 merged peaks.

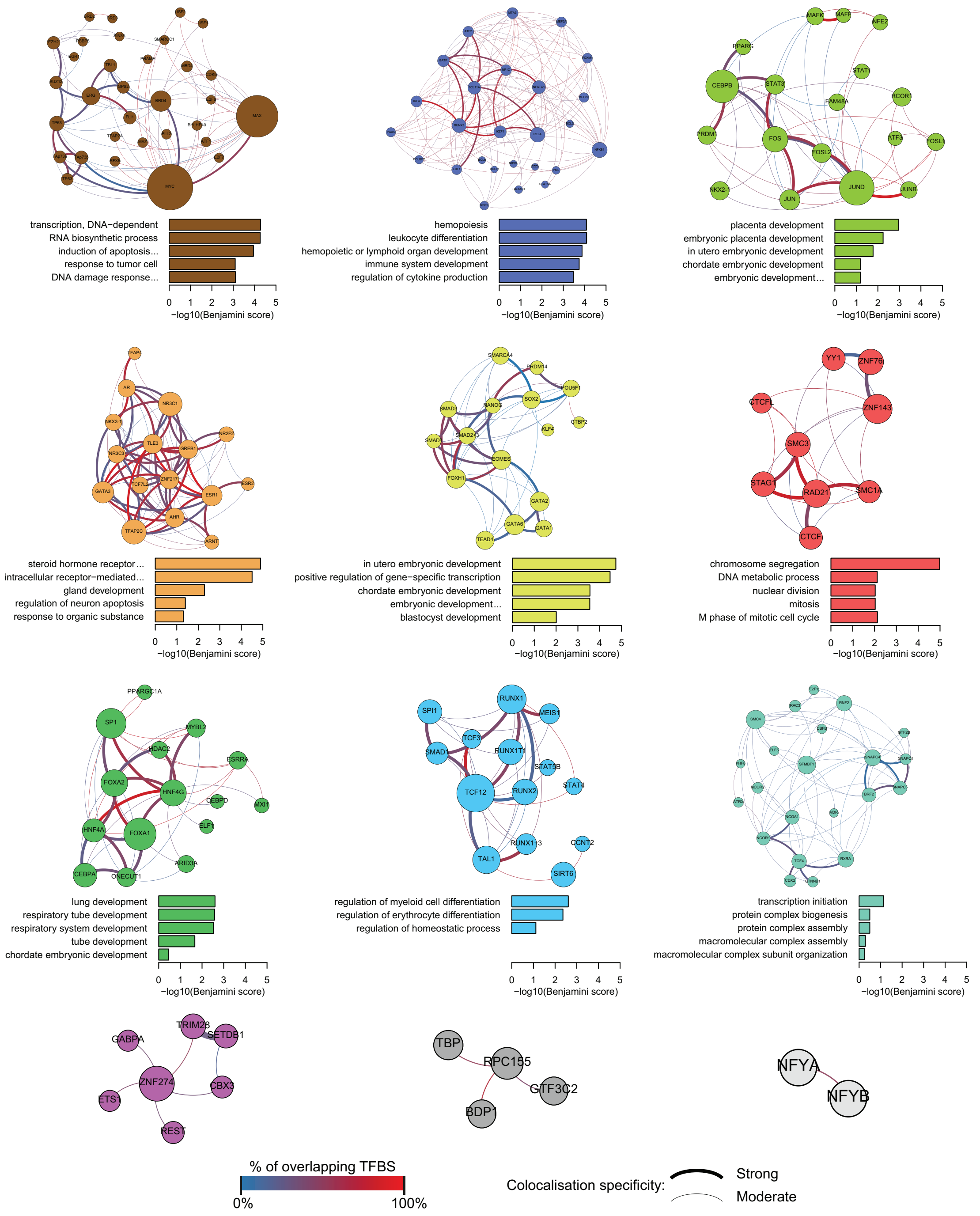

Figure S4 Clusters of highly interconnected and strongly specific colocalized TFs.

The colocalisation network in Figure 4 reveals 12 clusters of strongly specific TFs isolated using a partitioning algorithm. Nodes of these sub-networks indicate individual TFs, edge colours depict the percentages of overlap between TFBS and weights the colocalisation specificity between two TFs. Enrichments of sub-networks in Gene Ontology *Biological Process* annotations were calculated using DAVID tool and are indicated below each sub-network. No annotations were enriched in the last three sub-networks.

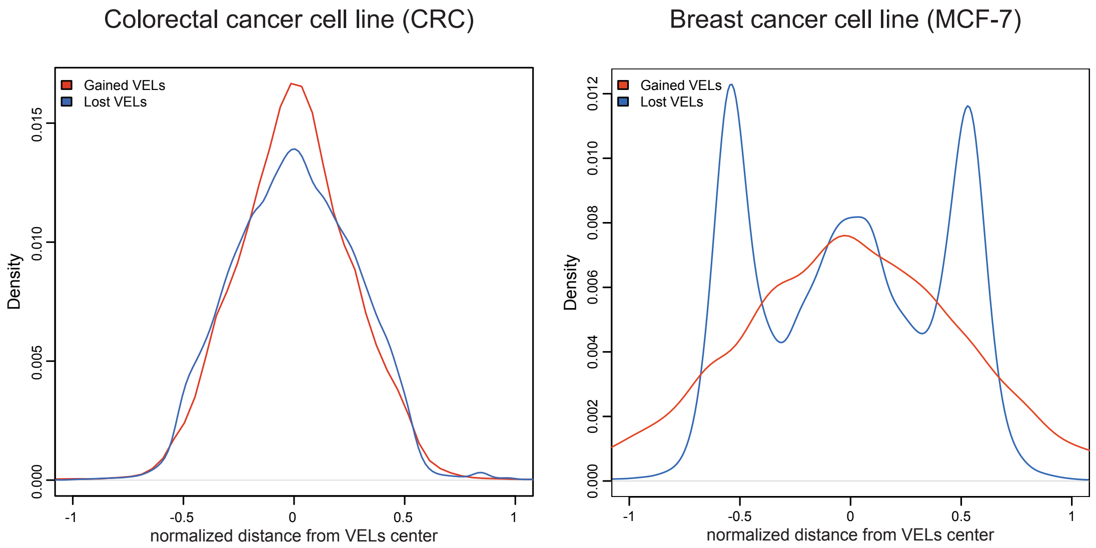

Figure S5 Normalized distance from TFBS to the center of Variant Enhancer Loci.

VELs overlapping with one or more binding sites have TFBS located preferentially at the center of those loci. Distances between TFBS and the center of VELs are plotted in a normalized scale taking into account the diversity of VELs size.

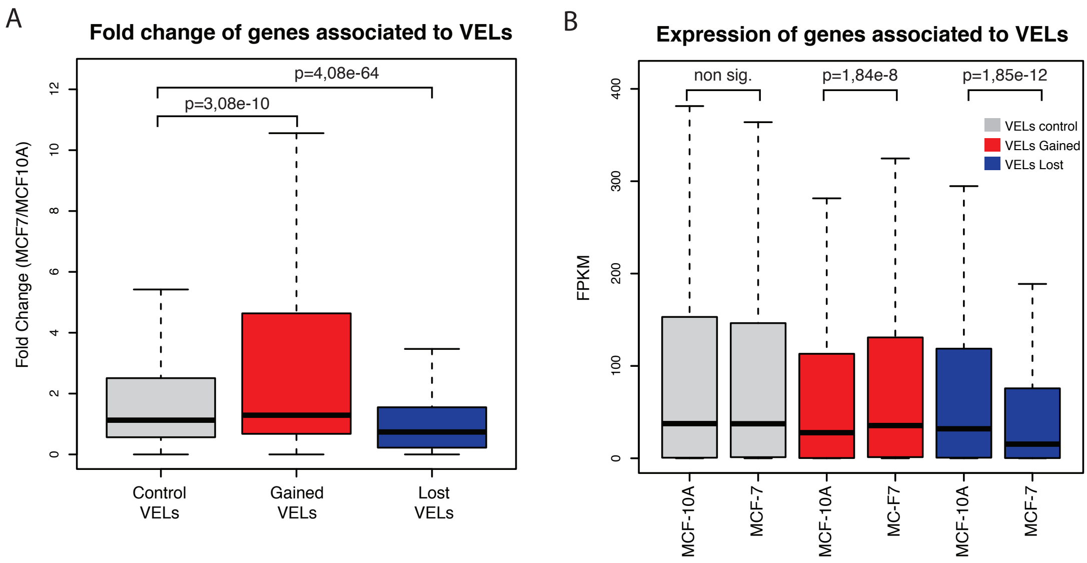

Figure S6 Breast cancer VELs correlate with gene expression variations.

(**A**) Gene expression fold changes associated with control, gained and lost VELs in MCF-7/MCF-10A cell line (GSE48213). (**B**) FPKM levels of genes associated with control VELs (gray) and aberrantly expressed genes associated with Gained (red) & Lost (blue) VELs in breast cancer (MCF-7) and normal mammary epithelial cell lines (MCF-10A).

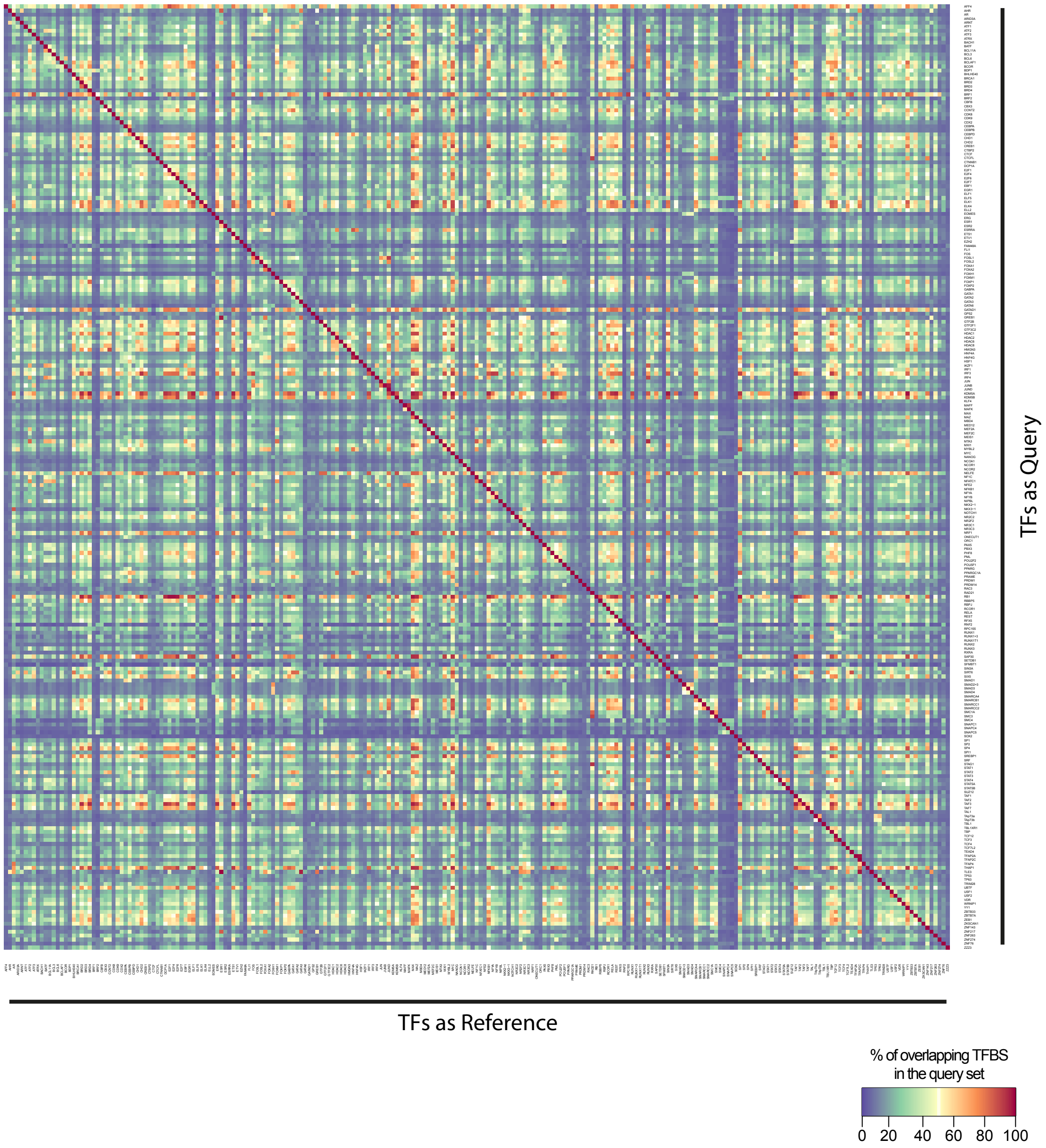

Figure S7 Matrix of the percentages of significant overlapping peaks for each pair of TFs.

In this figure we are computing the overlap of non-redundant binding sites for each couple of transcription factors using IntervalStats tool. The specificity of this analysis is that we used each TF both as a query and as a reference in each couple of TFs (eg; TFa vs TFb, TFb vs TFa). Thus, this analysis forms an asymmetric matrix of the percentages of significant overlapping peaks between two TFs. For a TF pair (TFa vs TFb) and for each peak in the query set TFa, IntervalStats computes a p-value of the overlap of a peak with the reference set of TFBS for TFb. We identified significant overlapping peaks with the reference TF with a p-value threshold defined at 0.05. The result is an asymmetric matrix of the percentages of significant overlapping peaks between two TFs. TFs used as queries by IntervalStats are indicated on the right side of the heatmap and TFs used as references at the bottom. The significant overlapping peaks in the query set of TFBS are indicated as percentages ranging from 0% of overlap (blue) to 100% of overlap (red). This asymmetric matrix allowed to determine a list of strongly and moderately specific TFs by identifying outliers based on the percentages of significant overlapping peaks (See Figure S8).

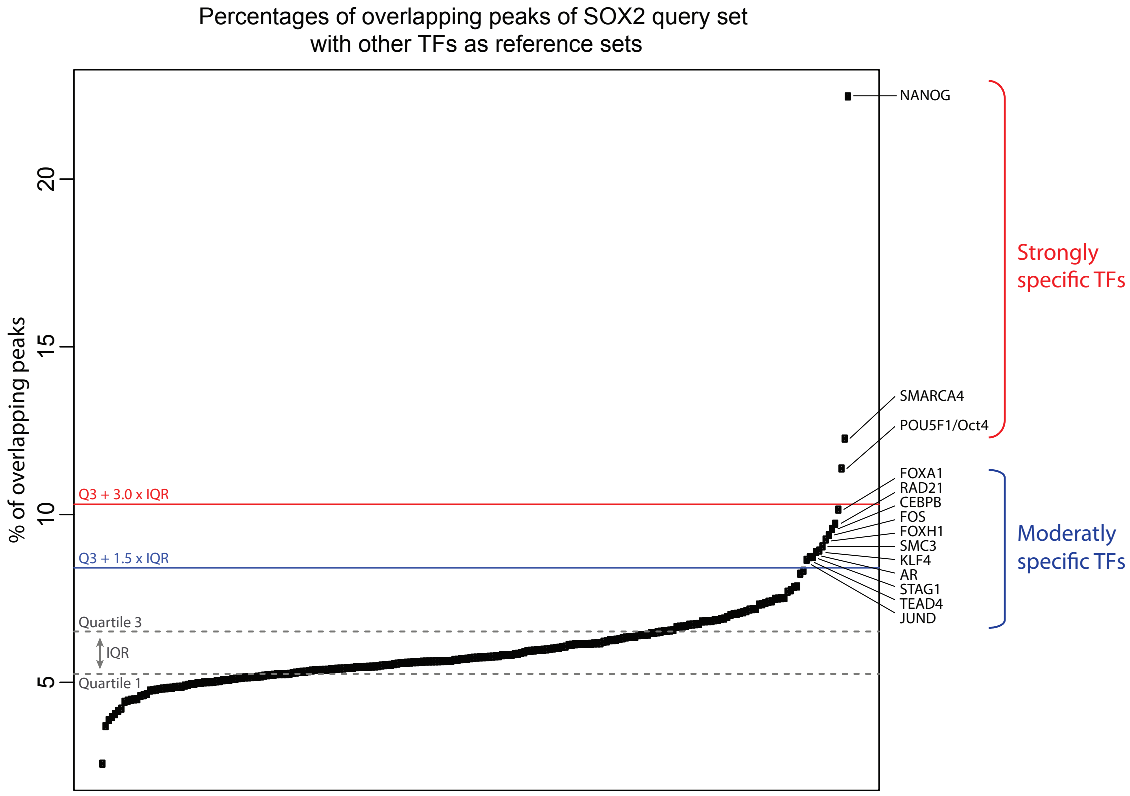

Figure S8 Determination of the colocalisation specificity of two TFs (example for SOX2).

For each transcription factor, a list of strongly and moderately specific colocalized TFs was determined by identifying outliers based on the percentages of significant overlapping peaks. Outliers were defined as TFs that have a percentage of overlap exceeding 1.5 and 3.0, respectively for moderately and strongly colocalized TFs, the interquartile range (IQR) above the 3rd quartile of percentages. For SOX2, three strongly colocalized TFs (NANOG, POU5F1/Oct4 and SMARCA4) and 11 moderately colocalized TFs (AR, CEBPB, FOS, FOXA1, FOXH1, JUND, KLF4, RAD21, SMC3, STAG1 and TEAD4) were identified.

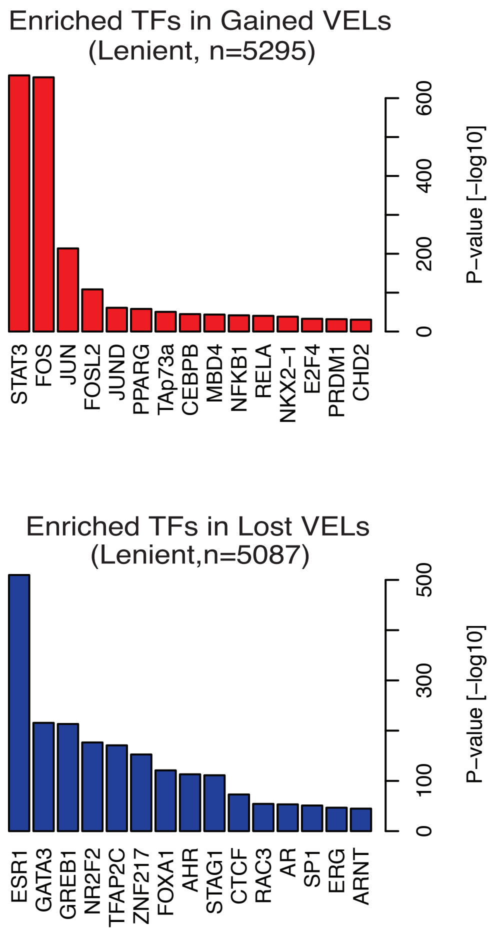

Figure S9 Transcription factors specifically enriched within lenient sets of gained and lost VELs in MCF-7.

To assess the MCF-7 VELs detection procedure we generated VELs for MCF-7 with relaxed thresholds (SD=0.75, n=5295 for Gain, n=5087 for Loss). Variant Enhancer Loci detections were performed using the exact same procedure as described in Akhtar-Zaidi B. *et al* using a function of Shannon Entropy. We observe a similar enrichment of TFs between our initial VELs (Figure 5c) and those lenient VELs where we can recapitulate 12 and 11 TFs out the 15 most enriched TFs for Gained and Lost VELs respectively.
